# Embryonic MicroRNA-369 Controls Metabolic Splicing Factors and Urges Cellular Reprograming

**DOI:** 10.1371/journal.pone.0132789

**Published:** 2015-07-15

**Authors:** Masamitsu Konno, Jun Koseki, Koichi Kawamoto, Naohiro Nishida, Hidetoshi Matsui, Dyah Laksmi Dewi, Miyuki Ozaki, Yuko Noguchi, Koshi Mimori, Noriko Gotoh, Nobuhiro Tanuma, Hiroshi Shima, Yuichiro Doki, Masaki Mori, Hideshi Ishii

**Affiliations:** 1 Department of Frontier Science for Cancer and Chemotherapy, Osaka University Graduate School of Medicine, Osaka, 565–0871, Japan; 2 Department of Cancer Profiling Discovery, Osaka University Graduate School of Medicine, Osaka, 565–0871, Japan; 3 Department of Gastrointestinal Surgery, Osaka University Graduate School of Medicine, Osaka, 565–0871, Japan; 4 Faculty of Science, Kyushu University, Fukuoka, 819–0395, Japan; 5 Kyushu University, Department of Molecular and Surgical Oncology, Tsurumihara 4546, Beppu, Ohita, 874–0838, Japan; 6 Division of Cancer Cell Biology, Cancer Research Institute of Kanazawa University, Kakuma-machi, Kanazawa, 920–1192, Japan; 7 Division of Cancer Chemotherapy, Miyagi Cancer Center Research Institute, Natori, 981–1293, Japan; National University of Singapore, SINGAPORE

## Abstract

Noncoding microRNAs inhibit translation and lower the transcript stability of coding mRNA, however miR-369 s, in aberrant silencing genomic regions, stabilizes target proteins under cellular stress. We found that *in vitro* differentiation of embryonic stem cells led to chromatin methylation of histone H3K4 at the miR-369 region on chromosome 12qF in mice, which is expressed in embryonic cells and is critical for pluripotency. Proteomic analyses revealed that miR-369 stabilized translation of pyruvate kinase (*Pkm2*) splicing factors such as HNRNPA2B1. Overexpression of miR-369 stimulated *Pkm2* splicing and enhanced induction of cellular reprogramming by induced pluripotent stem cell factors, whereas miR-369 knockdown resulted in suppression. Furthermore, immunoprecipitation analysis showed that the Argonaute complex contained the fragile X mental retardation-related protein 1 and HNRNPA2B1 in a miR-369-depedent manner. Our findings demonstrate a unique role of the embryonic miR-369-HNRNPA2B1 axis in controlling metabolic enzyme function, and suggest a novel pathway linking epigenetic, transcriptional, and metabolic control in cell reprogramming.

## Introduction

Cell metabolism plays a pivotal role in dictating whether a cell proliferates, differentiates, or remains undifferentiated. A profound biochemical feature that distinguishes cancer cells and induce pluripotent stem cells (iPSCs) from differentiated cells is their metabolic regulation, which is characterized by limited oxidative capacity and active anaerobic glycolysis [1]. Proliferative embryonic stem cells (ESCs) and cancer cells exhibit a high glycolysis rate, resulting in lactate production despite high oxygen levels. Recent studies suggest a critical role for epigenetics during stem cell differentiation compared with differentiated cells [2]. This involves upregulated expression of threonine dehydrogenase (TDH) in early blastocysts and ESCs as well as reprogramming of iPSCs [3, 4]. TDH and glycine dehydrogenase regulate 5-methyltetrahydrofolate synthesis, thereby modulating trimethylation of histone H3 lysine 4 (H3K4) [1]. H3K4 trimethylation is associated with open euchromatin, which is crucial for the epigenetic plasticity of PSCs and self-renewal through gene expression [5,6], indicating a close relationship between epigenetics and stem cell metabolism.

Micro RNAs (miRs) are a class of small noncoding RNAs that play critical roles in most developmental processes [7, 8] and diseases such as cancer [9–11]. Precursors, called primary miRs, formed after transcription are first processed in the nucleus into an intermediate precursor-miR (pre-miR) by enzymes such as Drosha and DGCR8 [12, 13]. Pre-miRs are then transported by the exportin 5-RanGTP shuttle to the cytoplasm for further processing by the ribonuclease type III enzyme DICER 1 into 22–24-bp mature miRs [14]. Mature miRs bind to the 3′-untranslated region (UTR) of target mRNAs via an imperfect match and regulate their translation and stability. This binding regulates the expression of more than 33% of protein-coding genes [15]. Although repression predominates [15], switching from repression to stabilization or activation reportedly upregulates miR subclass (e.g., miR-369) translation [16, 17]. This recruits Argonaute (AGO) and fragile X mental retardation-related protein 1 (FXR1) on the AU-rich element (AREs) and miR target sites [16, 17].

Ethical issues exist regarding the use of fertilized oocytes for ESC production as well as immunological compatibility with unrelated donors. However, a breakthrough addressing these concerns came with the discovery that full reprogramming can be achieved by introducing defined biological factors such as OCT4 (POU Class 5 Homeobox 11), SOX2, KLF4 (Kruppel-like factor 4), and c-MYC (v-Myc avian myelocytomatosis viral oncogene homolog) in mouse [18] and human [19] fibroblasts to generate iPSCs. Gene introduction for reprogramming events is often facilitated by adding miRs, which provide higher reprogramming efficiency [20–22]. A combination of histone deacetylase 2 (HDAC2) suppression and lentiviral-mediated transfection of immature miR-302/367 sequences is reported to activate OCT4 expression and induce reprogramming. iPSCs reprogrammed by miR-302/367 displayed similar characteristics (e.g., pluripotency, marker expression, and teratoma formation) to those reprogrammed using OCT4, SOX2, KLF4, and cMYC in mouse cells, including chimera and germline contribution [20]. Direct transfection of mature double-stranded miR (a combination of miR-200c, -302, and -369) led to PSC generation in both humans and mice from differentiated adipose-derived mesenchymal stem cells (ADSCs) [23]. This reprogramming method does not require vector-based gene transfer, which is suggestive of its significant potential in biomedical research and clinical settings.

The mechanisms underlying miR reprogramming are however not fully understood, yet efficient generation of qualified iPSCs is important for research. Electroporation of a polycistronic hsa-miR-302a/b/c/d cassette has reportedly led to human hair follicle cell reprogramming [22] through miR-302-targeted cosuppression of 4 epigenetic regulators. These regulators were AOF2 (also known as KDM1 or LSD1), AOF1, MECP1-p66, and MECP2 [22]. The retroviral polycistronic expression of hsa-miR-302a/b/c/d allowed formation of a PSC-like phenotype from human skin cancer cells [21]. Inhibition or reversion of epithelial–mesenchymal transition (EMT) was shown to be stimulated by miR-302 [20, 22, 24], -367 [20, 24], and -200c [23] during reprogramming, while TGF-β-mediated EMT signaling antagonized reprogramming. Furthermore, KLF4-stimulated E-cadherin expression, a hallmark of EMT, is an important reprogramming event, however the requirements of EMT inhibition may depend on cellular context [25]. The role of miR-369 encoded in aberrant silencing genomic regions on chromosome 12qF in mice [26] remains elusive.

## Materials and Methods

### Cell culture and transfection

All cancer cells were purchased from American Type Culture Collection (ATCC). Transfection was performed using the FuGENE-6 (Roche, Indianapolis, IN) transfection reagent, according to the manufacturer’s instructions, followed by lentiviral production in HEK-293T cells and viral infection (Roche, Tokyo, Japan). ESCs (v6.5 cell lines) were cultured in high-glucose DMEM (Nakarai, Kyoto, Japan) containing 15% FBS (Nitirei), 1 mM sodium pyruvate solution (Gibco, Tokyo, Japan), 1× MEM nonessential amino acids (Gibco), 0.1 mM 2-mercaptoethanol (Nakarai), and 1000 U/ml of ESGRO (Millipore, Billerica, MA, USA) on mitomycin treated MEF feeder cells in a CO_2_ incubator at 37°C.

### RNA analysis

Total RNA was extracted using phenol followed by precipitation (TRIzol kit, Invitrogen-Life Technologies Japan, Tokyo, Japan). cDNA was synthesized and quantitative real-time polymerase chain reaction (qRT-PCR) was subsequently performed using a Light Cycler (Roche). Amplified signals were normalized against GAPDH.

### Cell Reprogramming Assay

5 × 10^5^ mouse adipose derived mesenchymal stem cells were seeded in 100 mm dishes one day prior to infection. Retrovirus mixtures (*Oct4*, *Sox2*, *Klf4*, and *c-Myc*) were added to each dish and after 16–20 h, the virus solution was replaced with fresh medium containing puromycin (1 μg/ml). After 7 days, the cells were trypsinized and plated on MEF feeder cells with ESC medium supplemented with 15% FBS, 1 mM sodium pyruvate solution, MEM nonessential amino acids, 0.1 mM 2-mercaptoethanol, and 1000 U/ml of ESGRO. Cells were stained using an AP kit (Muto Pure Chemicals, Tokyo, Japan) 14 days after infection, and the number of AP-positive colonies was determined.

### Microarray experiments with miR

For miR microarray experiments, after assessment for quality, 500 ng of the extracted total RNA was labeled with Cyanine-3 (Cy3) using the Low Input Quick Amp Labeling Kit (Agilent). Dye incorporation and cRNA yield were assessed using the NanoDrop ND-2000 Spectrophotometer. The labeled RNAs were hybridized onto Agilent Mouse GE 8 × 60K Microarray for 17 h at 65°C in a rotating Agilent hybridization oven. After hybridization, microarrays were stringently washed for 1 min at room temperature with GE Wash Buffer 1 (Agilent, Tokyo, Japan) followed by GE Wash buffer 2 for 1 min at 37°C (Agilent, Tokyo, Japan) and then immediately dried by brief centrifugation. The fluorescent signals were then scanned with the Agilent DNA Microarray Scanner (G2565CA) and analyzed using Feature Extraction Software 10.10 (Agilent).

### Western blot analysis

Cells were lysed in a buffer containing EDTA-free protease inhibitors (50 mM HEPES [pH 7.5], 150 mM NaCl, 1% TritonX-100; Sigma Aldrich, Tokyo, Japan). Proteins were quantified using a protein assay kit (BioRad, Tokyo, Japan) and 20 μg of protein was subjected to SDS-PAGE, transferred to membranes. Antigens were then detected by probing with specific antibodies. All antibodies were purchased from Sigma Aldrich except for isoform-specific antibodies against PKM1 (rabbit polyclonal, Proteintech AP7476b, Chicago, IL, USA) and PKM2 (rabbit polyclonal, Proteintech 15821-1-AP, Chicago, IL, USA), which were generated using specific antigen-peptides. Blotting signals were quantified by image analysis (Multi Gauge Ver.3 FUJIFILM; Fuji, Tokyo, Japan). Mean and standard deviation of 3 independent experiments were determined.

### Immunoprecipitation

Samples were lysed by freeze-thawing in ice-cold lysis buffer (150 mM NaCl, 0.5% IGEPAL CA-630, 50 mM Tris-HCl, pH 8.0) containing Protease Inhibitor Cocktail (Complete EDTA-free, Roche). For complete disruption, the lysates were passed through a 22-gauge needle and centrifuged at 12,000 G for 10 min at 4°C to remove insoluble matter. The supernatants were subjected to immunoprecipitation with primary antibodies using Protein G Sepharose 4 Fast Flow (GE, Tokyo, Japan) according to the manufacturer’s protocol. Immune complexes were dissociated with SDS-sample buffer at 95°C for 5 min and subjected to SDS-PAGE analysis. Western blot signals were detected by ECL Prime Western Blotting Detection Reagent (GE) and hyperfilm ECL.

### Immunocytochemistry

Cells were fixed with 4% paraformaldehyde for 30 min at room temperature and then treated successively with 0.3% Triton X-100 (Wako Chemical; Osaka; Japan) in phosphate-buffered saline (PBS; Sigma) for 15 min followed by 3% bovine serum albumin (Sigma) for 30 min to reduce nonspecific reactions. Samples were allowed to react overnight with anti-PDX1 (Abcam ab47308) antibodies at a 1:200 dilution at 4°C. They were then stained with Alexa Fluor 488 conjugated anti-guinea pig IgG antibody (ab150185; Abcam, Tokyo, Japan) as the secondary antibody for 1 h at room temperature. Their nuclei were stained with DAPI for 10 min. Images were acquired on a fluorescence microscope (Keyence BZ-9000).

### ChIP-PCR Assay

Cells were trypsinized and homogenized in 10 volumes of PBS, resuspended in 1% formaldehyde dissolved in PBS, and cross-linked at room temperature for 5 minutes. The reaction was stopped by adding glycine (0.2 M). The cells were washed twice with cold PBS and resuspended in ice-cold cell lysis buffer (10 mM NaCl, 10 mM Tris-HCl pH 8.0, 0.5% NP-40). Nuclei were washed with cell lysis buffer and resuspended in nuclear lysis buffer (1%SDS, 10 mM EDTA, 10 mM Tris-HCl pH 8.0). The samples were rotated for 10 minutes at 4°C and added to ChIP buffer (50 mM Tris-HCl pH 8.0, 167mM NaCl, 1.1% TritonX-100, 0.11% Sodium Deoxycholate, protease inhibitor mix). Chromatin was sonicated to 300–500 bp, followed by standard ChIP analysis with the following antibodies: histone H3K4 unmodified (Millipore, 05–1341), histone H3K4 monomethyl (Millipore, 07–436), histone H3K4 dimethyl (Millipore, 05–1338), histone H3K4 trimethyl (Millipore, 05–1339), histone H3K9 trimethyl (Abcam, ab8898), histone H3K27 trimethyl (39155; Active Motif, Tokyo, Japan), and histone H3K79 dimethyl (Abcam, ab3597). Primer sequences used were as follows: position 1 (5′-ACCCTTGGGAAGCTAGGATT-3′ and 5′- ACGGAGGACACTTGGACTCT-3′); position 2 (5′-GCTTTTTAATGGCTGTGCATATAC-3′ and 5′- AGCAATTAATCTCACTAGTGCCT-3′); position 3 (5′-GCTTACGACAACCGACAACA-3′ and 5′-TAAGCAAGGGCTGCTCAGAT-3′); position 4 (5′-CAGTGGAAATCCCCATCAAC-3′ and 5′-GCTGAGAGCTGGATTGGTGT-3′); position 5 (5′-GTCCCATTGGTAAGCTGGTG-3′ and 5′-CTGTGGTCACCTGAACCTGA-3′); position 6 (5′-CTAACCCATGCGAGAACGAT-3′ and 5′-GCTTGCACAGACACTCGAAG-3′); position 7 (5′-ACCAGGAGTCAAGCGACAGT-3′ and 5′-GCTGACCTTCCTCTTCACCA-3′); position 8 (5′-GGTCTTCAGGCAGTTGCATT-3′ and 5′-TGGCAGTGGTCATCTTTGAG-3′); position 9 (5′-ACAGCTCTGGAGGTTGGTGT-3′ and 5′-GAGGAACTCTGCAGCTCTGG-3′); position 10 (5′-ATCGTGGTCCTCTGGACAAG-3′ and 5′-ACCTTCCAGTATGCGGTCAG-3′); position 11 (5′-GGGAATTGCCAAGTCTGAAG-3′ and 5′-GCATGGAGACCAAATATCCA-3′); position 21 (5′-AGGTCTGAAGGTCACGTTGG-3′ and 5′-CCTGAGACTGGGATCTGGAC-3′); position 22 (5′-CTTCCTGGGTGACATGATCC-3′ and 5′-CAGGCAGAGGAAACAGAATG-3′); position 23 (5′-GTCGACACAAGCACAAGCTC-3′ and 5′-AGGAAGACGACGGATAGCAC-3′); position 24 (5′-GACCGTCATCGCATCTGTC-3′ and 5′-GAGAGACCACACCCTTCTGC-3′), position 25 (5′-CCGAGCTTTGGTACTTGGAG-3′ and 5′-TGTCCAAGGATGTGTGATGG-3′); position 26 (5′-GGATGCTGGTATTCCTGCTG-3′ and 5′-CAGGTAAGGCTAACCCATGC -3′); position 27 (5′-TTCCTCTTGGCGCTTATTTG-3′ and 5′-CAACCTCCCTCCAAGTACCA-3′); position 28 (5′-CAAGCTCCTGTGGCTTCTTC-3′ and 5′-GCAACCTCCATTCGAACACT-3′); position 29 (5′-CTTCTGGAAGGCATCGTCTC-3′ and 5′-CACCCTTTCACCTTCCAAGA-3′); position 30 (5′-TTGACTCCAGAAGATGCTCC-3′ and 5′-CCTCAGGTTCCTAAGCAAGG-3′); and Gapdh (5′-CTGTACGGGTCTAGGGATGC-3′ and 5′-CCGACCTTCACCATTTTGTC-3′).

### Protein stability assay

All fragments of HNRNPA2/B1 cDNA were synthesized (Genescript) and cloned into a retroviral pMXs-IP vector (Cell Biolabs, San Diego, CA, USA). Cells were grown at a density of 1×10^5^ cells/well in 6 well plates. After 24 hours, lentivirus mixtures (*Oct4*, *Sox2*, *Klf4*, and *c-Myc*) were added to each well. After another 24 hours, the medium was replaced with fresh medium and lentivirus-miR-369 and retrovirus of HNRNPA2/B1 deletion mutant vectors were added to each well. The samples were subjected to analysis on day 5. For the run-on assay, 2×10^5^ cells were used as above except that miR-369 was introduced using lipofectamin RNAi Max (Life Technologies, 13778030). On day 3, medium containing 10 μg/ml of cycloheximide (CHX; Sigma, C4859) or 2 μg/ml of actinomycin D (ACD; Sigma, A9415) was added and samples were analyzed at 0, 2, 4, 6, 10, and 24 h.

### Statistical Analysis

Categorical variables were compared using the chi-square test. Continuous variables (medians/interquartile ranges) were compared using the Wilcoxon test. Statistical analyses were performed using JMP (JMP version 8.01, SAS Institute, Cary, North Carolina). *P* values of <0.05 were considered statistically significant.

## Results

### Role of miR-369 in the *DLK1-DIO3* gene cluster

**Fig 1:**
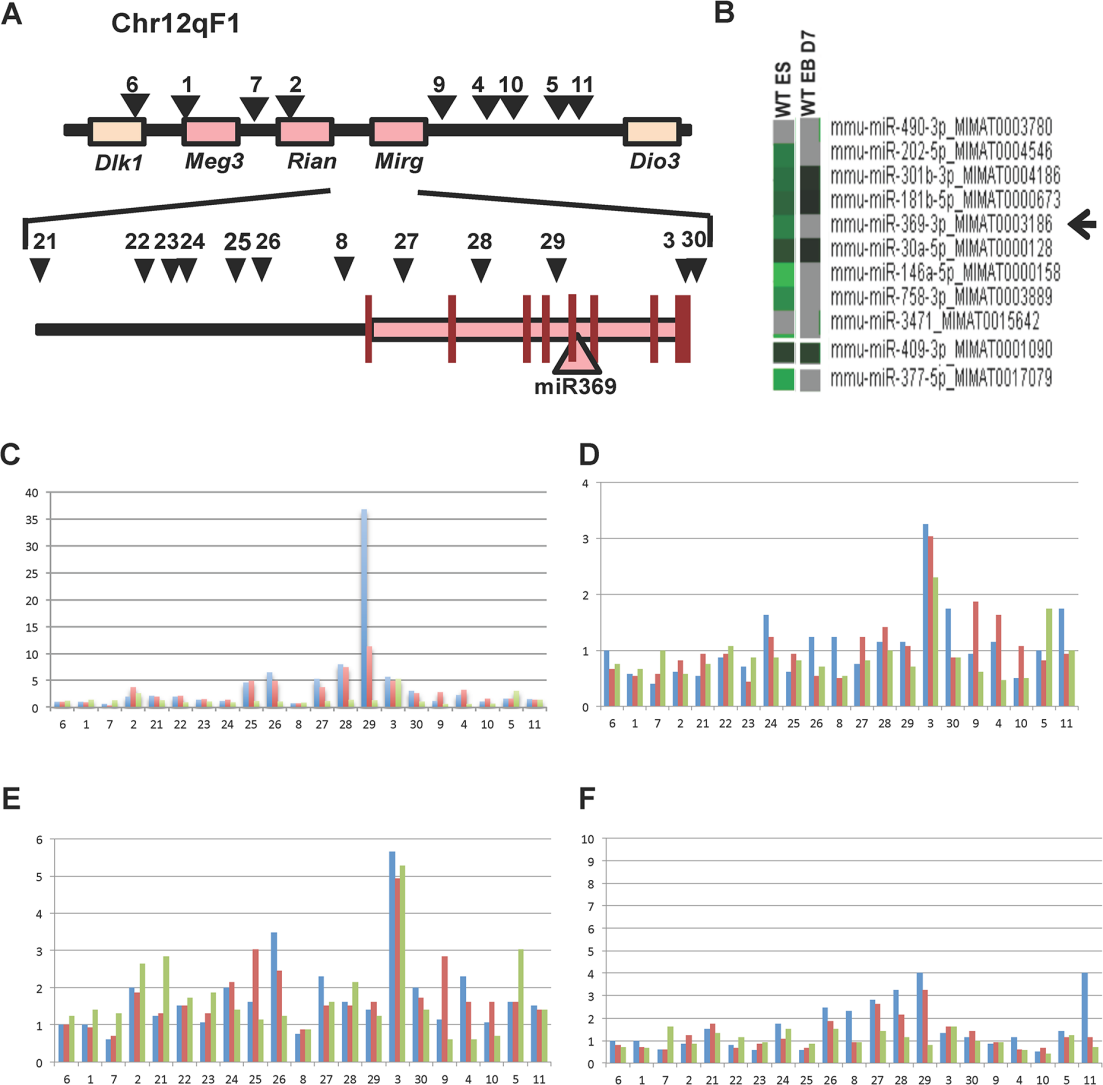
Role of transcripts located at mouse chromosome 12F1 in *Oct4* deprivation-induced differentiated ESCs. A) Mouse genomic regions involving *Dlk1*, *Meg3*, *Rian*, *Mirg*, and *Dio3*. miR-369 locations and ChIP-PCR primer positions are shown. B) Microarray analysis of miR expression level in undifferentiated ESCs (WT ES) and differentiated ESCs (WT EB D7). C) H3K4 tri-methylation in ESCs is shown at various time points indicated as: day 0 (Blue), 3 (Red), and 7 (Green). D) H3K9 tri-methylation in ESCs is shown at various time points indicated as: day 0 (Blue), 3 (Red), and 7 (Green). E) H3K27 tri-methylation in ESCs is shown at various time points indicated as: day 0 (Blue), 3 (Red), and 7 (Green). F) H3K79 tri-methylation in ESCs is shown at various time points indicated as: day 0 (Blue), 3 (Red), and 7 (Green). Vertical axis indicates relative amount of DNA.

Comparing genetically identical mouse ESCs and iPSCs by genome-wide search, it was reported that the overall mRNA and miR expression patterns of both cell types were indistinguishable, except for a few transcripts encoded in aberrant silencing genomic regions within the imprinted *Dlk1-Dio3* gene cluster on mouse chromosome 12qF [26]. Accordingly, we initially compared human and mouse genomic sequences and observed that *Rian* and *Mirg* were encoded on mouse chromosome 12qF (Fig 1A; Fig A in S1 File), but not in the analogous human chromosome 14q32.2–q32.31 (Fig B in S1 File). *Mirg*, but not *Rian*, harbors a partially homologous region (Fig C-E in S1 File) and contains miR-154, -377, -541, -409, -410, and -369 s, which are differentially expressed in ESCs and iPSCs [23, 26].

To assess the expression level of miR369 in pluripotent and differentiated cells, we performed a microarray analysis on undifferentiated and differentiated ESCs that were induced by embryoid body (EB) formation for a week. High expression of miR369 was observed in pluripotent ESCs, however this was decreased in differentiated cells (Fig 1B). Subsequently, to assess the epigenome of the miR369 coding region (chromosome 12qF1), we performed a ChIP–PCR analysis. It is known that methylated H3K4 and H3K79 are active chromatin markers, whereas methylated H3K9 and H3K27 are inactive. We therefore performed the ChIP–PCR analysis with antibodies against H3K4me3, H3K9me3, H3K27me3 and H3K79me3 in undifferentiated ESCs (day 0) as well as differentiated ESCs induced by EB formation for 3 days or a week (Fig 1C–1F). Each PCR primer position is shown in Fig 1A. The data demonstrated that in undifferentiated ESCs, the miR369 coding region was highly methylated at H3K4, H3K9, H3K27 and H3K79, but methylation at the active marks such as H3K4 of the miR369 promoter (primer position 29 in Fig 1C) was rapidly decreased and depended on EB formation. These results suggest that the miR369 region retains a bivalent chromatin status, which can be altered dynamically according to ESC differentiation. The findings also support the notion that H3K4 trimethylation in the *Mirg* locus of the *Dlk1-Dio3* gene cluster activates miR-369 promotion early in differentiation, and therefore the miR-369 region plays a role in the induction and maintenance of the pluripotent state in ESCs. Not only is this subjected to aberrant silencing by imprinting in order to distinguish the full developmental potential of mouse ESCs [26], but miR-369 introduction elicits reprogramming in humans and mice [23]. The focus of the present study is therefore to elucidate the exact role of mIR-369.

### The role of miR-369 in controlling the pluripotent state

**Fig 2:**
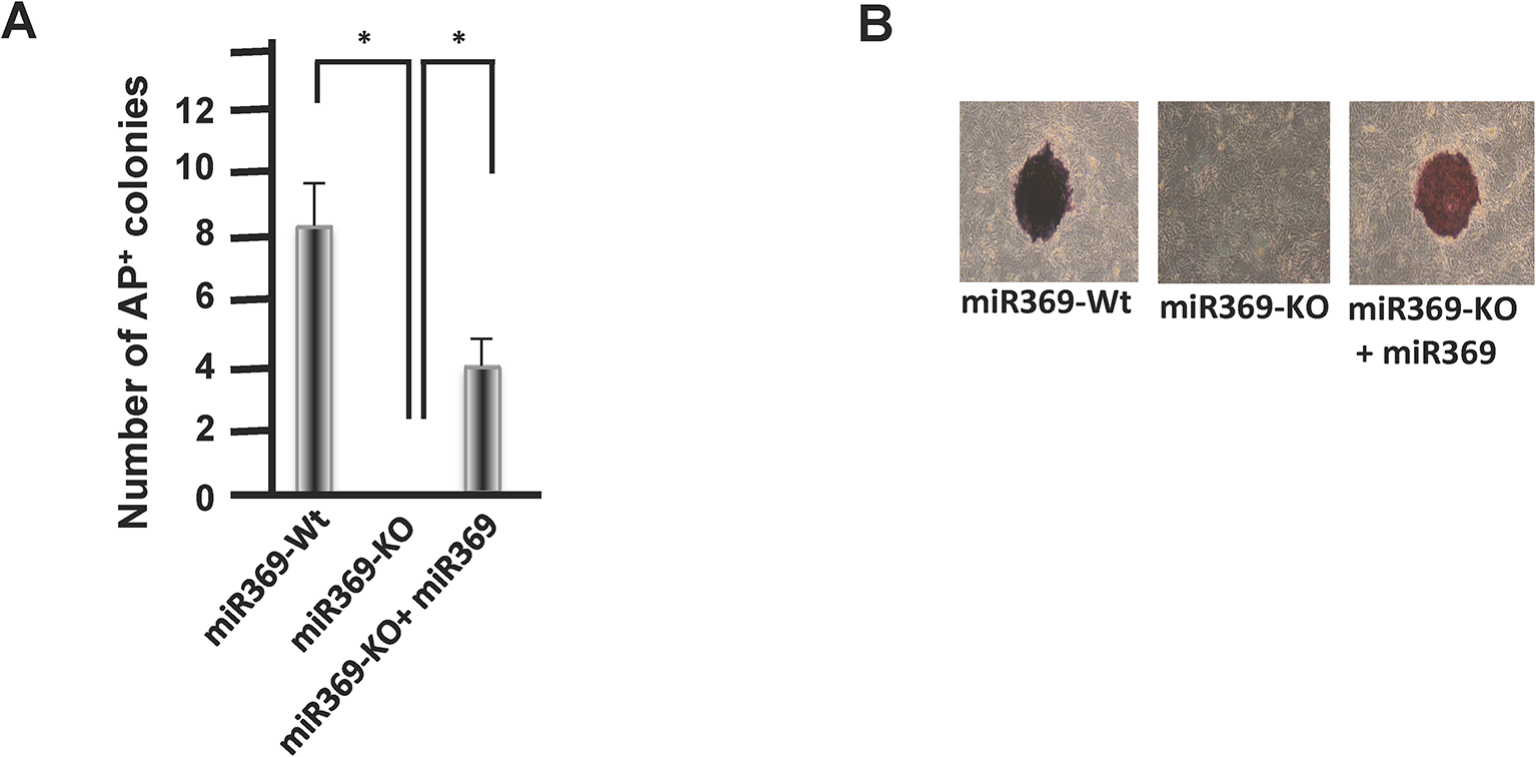
The effect of miRs on cellular reprogramming. A)Number of colonies formed with a typical iPSCs phenotype. The miR369-KO ADSCs were isolated from miR-369 KO mice. B) Alkari phosphatase stain in reprogrammed iPSCs.We confirmed the reproducibility of results by performing experiments.

There are over 1,500 miRs that exert critical physiological and pathophysiological roles in humans [27]. To confirm the role of miR369 in maintaining pluripotency and increasing cellular reprogramming efficiency, as seen with defined factors [20–22], we tested reprogramming in miR369 deficient ADSCs. The miR369 knockout resulted in absence of reprogramming, which was significantly rescued by expression of exogenous miR369 (Fig 2A and 2B), suggesting that miR369 may be involved in the early stages of reprogramming. These data demonstrate that miR-369 plays a critical role in cellular reprogramming.

### Splicing factors (SFs) for pyruvate kinases are controlled by miR-369

**Fig 3:**
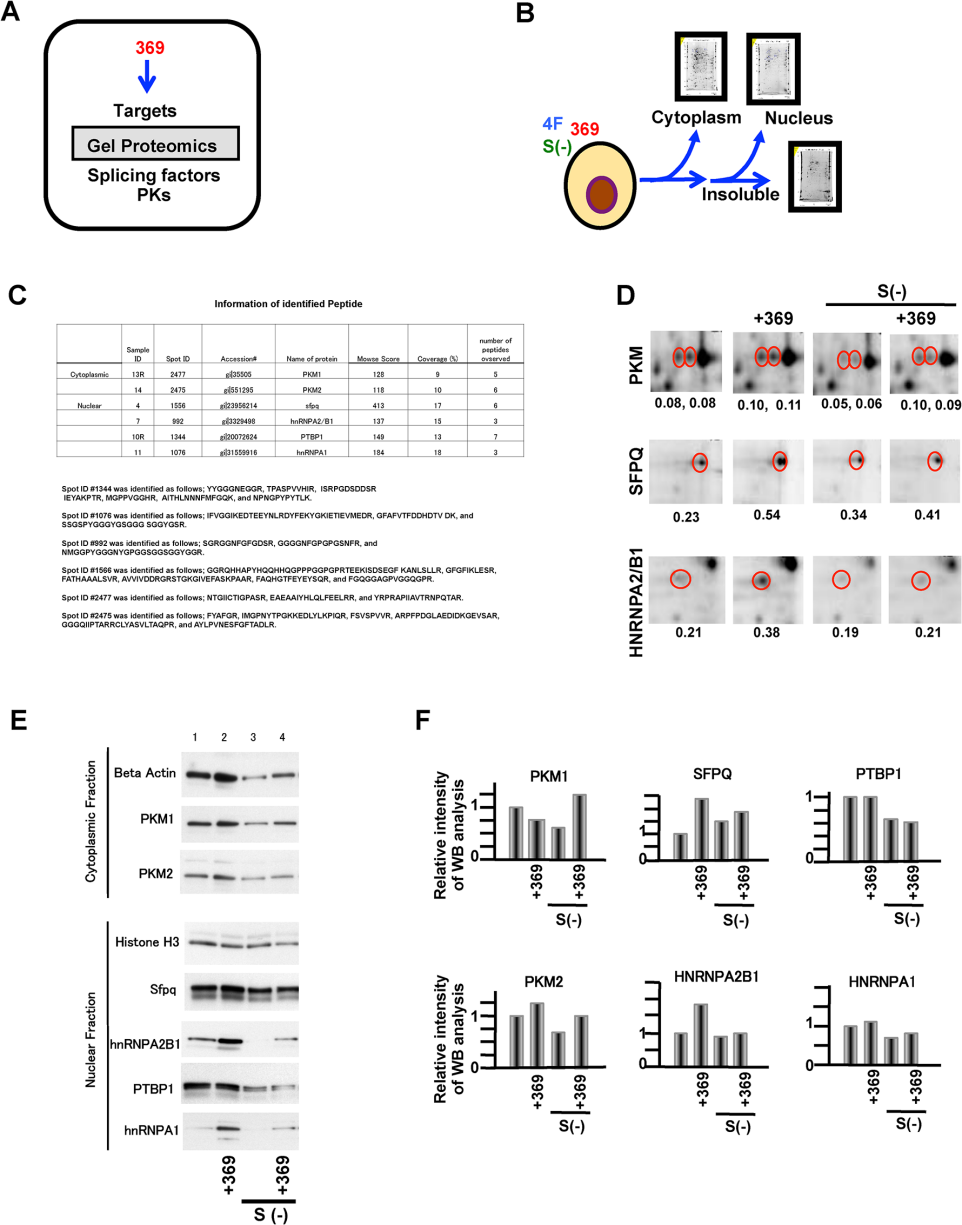
Proteomic identification of miR-369-downstream targets and the pathway study. **A)** Schema of Fig 4B–4E. PKs = pyruvate kinases. B) Whole gel proteomic study. miR-369 was introduced and cell lysates were separated into cytoplasmic, nuclear, and insoluble fractions, and subjected to the study. S(−) = serum free; 4F = transfection with Oct4, Sox2, c-Myc, and Klf4. C) Identification of indicated targets by 2-D gel cytoplasm (nucleus; insoluble, in parenthesis). All extractable proteins were subjected to peptide sequencing. D) Representative gel proteomic data. Signal intensity (arbitrary unit) is shown per protein. The mass spectrometry analysis indicated that each spot in the top panel contains both PKM1 and PKM2. E) Western blot analysis of miR-369 over expressed ADSCs. F) Quantitative study of (E).

**Fig 4:**
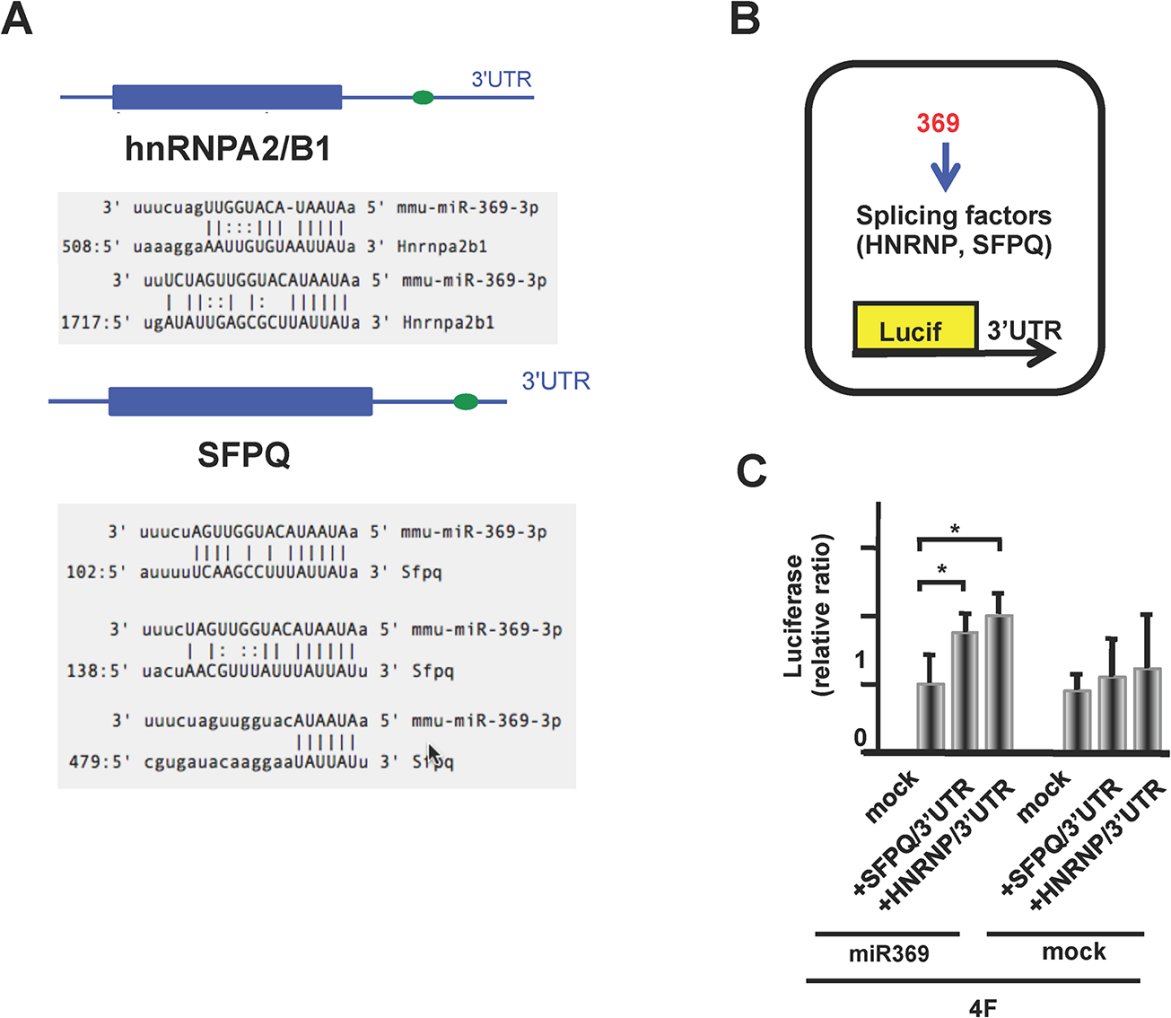
The miR-369 binding sequence and targeting effect. **A)** A schematic representation of HNRNPA2B1 and SFPQ mRNAs. Shaded areas represent the miR-369 binding sequences. B) Schema for (C). C) Effect of miR-369 on the 3′-UTR of HNRNPA2/B1. ADSCs transfected with 4F were subjected to luciferase chemiluminescence. Luminescence per *Luc* transcript measured by qRT/PCR was determined.

Given the recent reports that miR-369 upregulates translation [16, 17], we analyzed the proteins that were increased during miR-369-dependent cellular reprogramming using protein gel proteomic analyses by 2-D electrophoresis. Whole proteins were separated into 2,985 (cytoplasm), 1,680 (nuclear), 1,573 signals (insoluble) from miR-369 and 4Fs (Oct4, Sox2, c-Myc, and Klf4) transfected murine ADSCs [23]. By compared with ES cells, signals which were expressed just in fibroblasts or hepatocytes were excluded and subjected to the mass spectrometry analysis. This approach allowed the identification of gel spots showing increases in miR-369 and 4Fs transfected ADSCs and mouse ESCs compared with fibroblasts and hepatocytes (Fig 3A and 3B). The results showed an increase of 6 spots in miR-369 and 4Fs transfected ADSCs and in ESCs, but not in differentiated fibroblasts and hepatocytes. The proteins were identified as: hnRnpa2/b1, SFPQ, PTBP1, HNRNPA1, PKM1, and PKM2 using MS spectrometry (Fig 3D and 3E). We confirmed the results by immunoblot using ADSCs transfected with the 4Fs (Oct4, Sox2, c-Myc, and Klf4) and miR-369 and observed increases in hnRnpa2/b1, SFPQ, PTBP1, and PKMs (Fig 3F and 3G). We then examined whether the mRNAs have miR-369 binding sites in their 3’UTR using the following databases: http://www.microrna.org, http://www.targetscan.org. Our findings demonstrated that miR-369 binds the 3’UTRs of hnRnpa2/b1 and SFPQ mRNAs, however there were no miR-369 binding sites identified on PTBP1 or PKMs (Fig 4A). At peak endogenous miR-369 expression, miR-369 and reporter vector co-transfection demonstrated that exogenous miR-369 increased HNRNPA2/B1 and SFPQ, as determined by luciferase reporter activity, compared to control cells without miR-369 transfection (Fig 4B and 4C). The ratio of luciferase activity per LUC transcript showed stable translation under exogenous miR-369 expression. In contrast, mock transfection did not increase luciferase activity, suggesting an efficient increase in protein translation through the 3′-UTR of HNRNPA2/B1 and SFPQ.

### The miR-369–PK axis controls anaerobic glycolysis in PSCs

**Fig 5:**
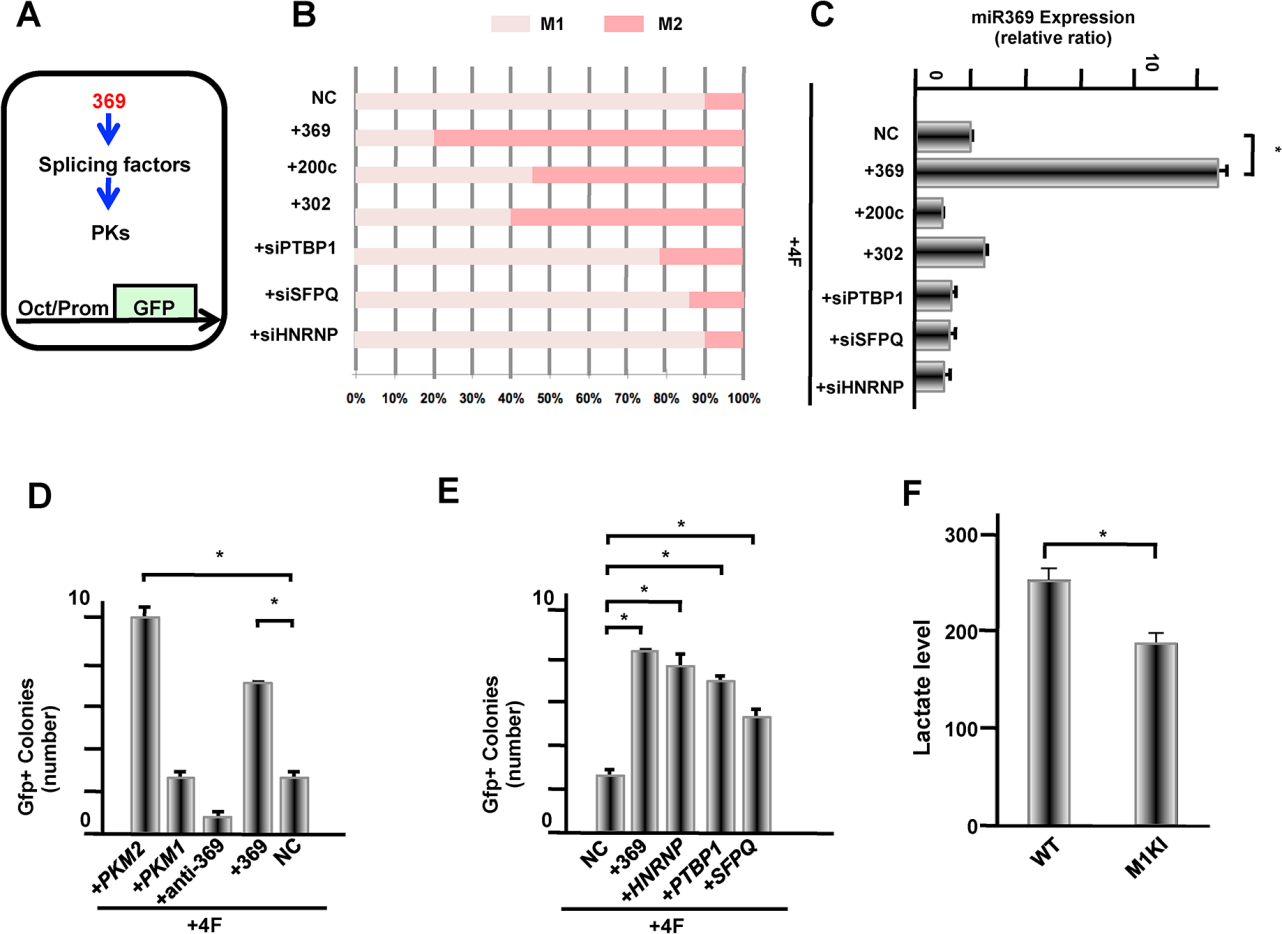
Identified miR-369 targets and their effect on cellular reprogramming induction. **A)** Schema of Fig 5B–5F. Role of the miR-369–PK pathway on cellular reprogramming. B) Ratio of PKM1 and PKM2 transcripts, measured by qRT-PCR with specific primers. The ratio of each transcript to total PK is shown (%). C) miR-369 transcript introduced by qRT-PCR. D, E) Number of reprogramming colonies. The experiment was performed three times and showed reproducibility. F) Quantification of the lactate levels. Wt = undifferentiated ESCs that mainly expressed PKM2; +PKM1 = PKM1 overexpressed ESCs.

To address if splicing of the PKM isoform of *Pkm* (*Pkm2*) was critical for cellular reprogramming, the 3 miRs (miR-200c, miR -302, or miR -369) were transfected (Fig 5A–5C). Given that miR200c [23] and miR -302 [20, 22, 24] are involved in the induction of reprogramming, we hypothesized that transfection with these miRNAs could elicit cellular responses at the transcriptome and metabolome levels during the reprogramming process. As expected, the *Pkm2* splicing isoform was significantly increased in miR200c and miR-302 transfected cells, however the effect was much more apparent in miR-369 transfected cells (Fig 5A and 5B), suggesting that miR369 directly targets SF expression of Pkm2. *Pkm2* splicing was suppressed by inhibition of endogenous SF expression (HNRNPA2B1, PTBP1, and SFPQ) using small interfering RNAs (siRNAs), to a degree similar to the mock control, and this was confirmed by noticeable PKM2 inhibition. Quantitative analysis of PKM1 and PKM2 showed an inhibitory effect of *Pkm2* splicing by siRNA inhibition of endogenous SFs (Fig 5B and 5C). To elucidate the significance of the miR-369 pathway for cellular reprogramming, we measured GFP^+^ colonies by OCT4-promoter driven GFP transgenic ADSCs. Similar to a previous study [28], we show that transfection of PKM2, but not PKM1, cDNA, led to an increase of GFP^+^ colonies (Fig 5D). Likewise, exogenous miR-369 expression, but not miR-369 inhibiton by anti-miR-369, also increased the GFP^+^ colonies. Furthermore, exogenous SF expression (HNRNPA2/B1, PTBP1, and SFPQ) also increased GFP^+^ colonies (Fig 5E). Therefore, the miR-369 pathway at least partially stimulated cellular reprogramming via the function of SFs. To confirm the effect of miR-369 on metabolism, we measured the lactate level of cultured PKM1 knock-in ADSCs using ELISA [29]. The results indicated that lactate level was decreased (Fig 5F). The data was consistent with the metabolome study in whole wide (Fig A, B in S3 File), showing that amount levels of numerous metabolites were altered between PKM1 knock-in and Wt cells [29]. We then studied the lactate level of cultured miR369 transfected ADSCs. The data showed that lactate level was increased in a time dependent manner of culture, but did not show significant differences between miR-369 and Wt (Fig C in S3 File.), suggesting a long term exposure may be necessary to induce metabolic changes. Recently, we reported that PKM1 plays an important role in the differentiation of ESCs, whereas PKM2 may modulate oxidative phosphorylation to maintain pluripotent ESCs [29]. PKM function may therefore define a branching status of ESC differentiation into each specific lineage. The present data confirm that miR-369 stabilizes the SFs of PKM2 thereby increasing biosynthesis, an important biological process for ESCs and presumably cancer cells [30, 31].

### *HNRNPA2/B1* translation in RNA-induced silencing complex is stabilized by miR-369

Previously miR function was shown to require RNA-induced silencing complex (RISC) assembly, which comprises small RNA and the Ago proteins [32]. How miR-369 controls the HNRnpa2/b1 in RISC is still not fully understood. The present study shows that culture in 0.1% serum medium or 4F stimulated luciferase activity showing the reporter gene expression at the transcriptional and translational levels (Fig 4C), suggesting a mechanism similar to previous reports, involving recruiting AGO and FXR1 on AREs in low serum conditions [16, 17]. AGO proteins play multiple roles in post-transcriptional regulation in animal cells, and repress gene expression by inducing mRNA degradation by RNAi and non-RNAi mechanisms or by translational arrest. Conversely, the effects of AGO proteins are modulated by specific cellular conditions such as HuR (an AU-rich-element binding protein)-mediated relief of repression [33], the stimulatory effect of AGO2/FXR1 on translation [16, 17], and the stimulatory effect of miR-122 on RNA-replication of the hepatitis C virus [34].

**Fig 6:**
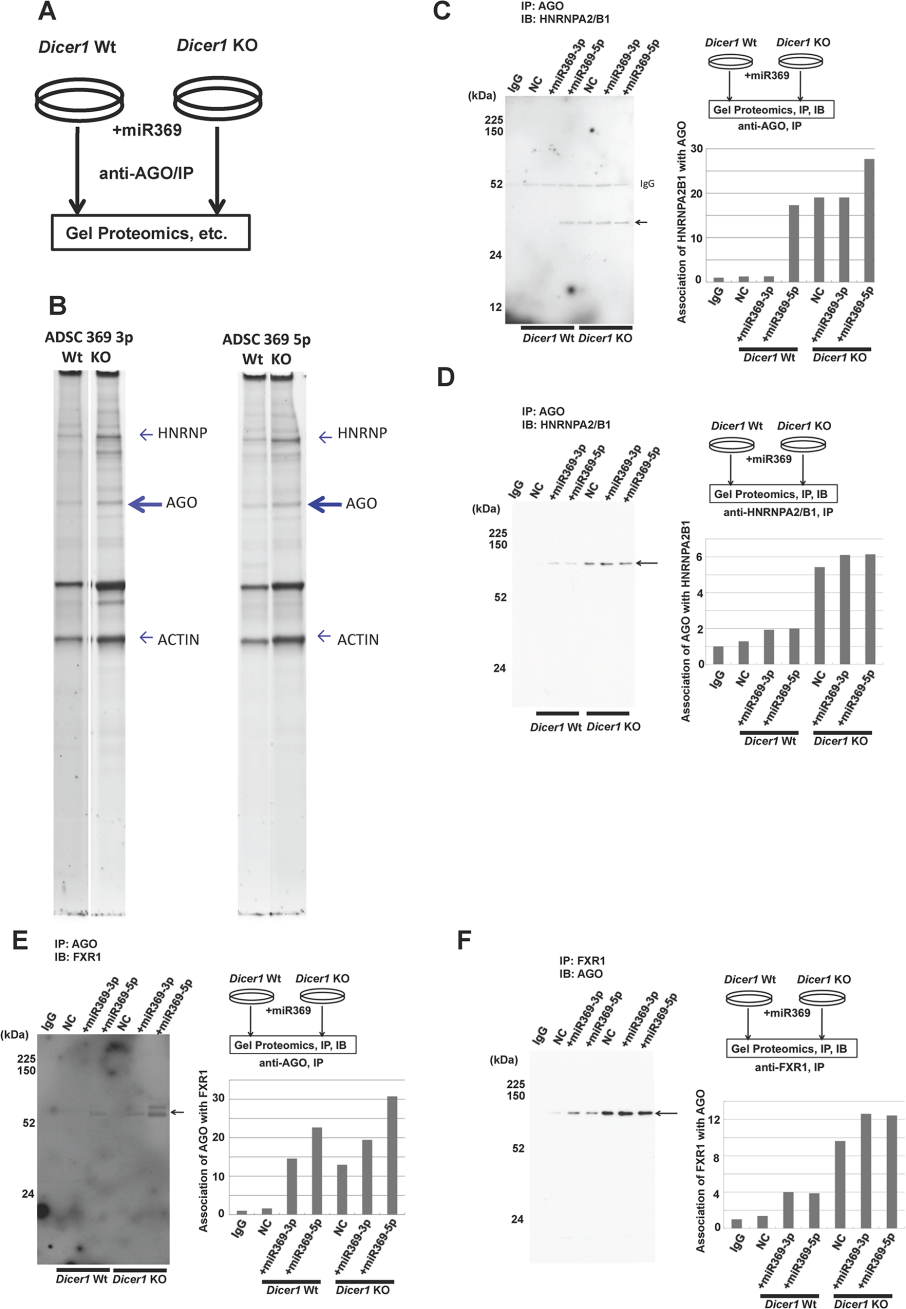
Characterization of miR-369-containing AGO complex. A) Schema of Fig 6B–6E. B) Characterization of miR-369-containing the AGO1 complex. Silver staining and LC–MS analyses allowed hnRnpa2/b1 identification, which was apparent in *Dicer1*-knockout (KO) ADSCs after miR-369 transfection. C, D, E, F) Association of AGO1 and hnRnpa2/b1 was confirmed by immunoprecipitation with each antibody, followed by western blot with the counterpart antibody in miR-369 transfected *Dicer1*-KO ADSCs. Controls = miR-369-5p and Dicer1 wild-type (Wt) ADSCs.

Since we are interested in factors involved in translation stabilization under reprogramming, we performed a co-immunoprecipitation experiment to detect proteins with miR-369 introduced under miR-depleted conditions in *Dicer*-deficient cells (Fig 6A). RISCs were extracted from *Dicer*-deficient ADSCs with or without miR-369 transfection and subjected to gel-proteomics. Interestingly, tandem mass spectrometry (MS/MS) analysis revealed that AGO was co-immunoprecipitated with HNRnpa2/b1 (Fig 6B) with strong association observed in *Dicer*-deficient cells, which could be stimulated by miR-369 (confirmed by immunoblot; Fig 6C and 6D). Previous reports have demonstrated the stimulatory effect of AGO2/FXR1 on translation [16, 17]. We therefore assessed their possible involvement and observed that miR-369 stimulated an augmented association under *Dicer*-deficient conditions (Fig 6E and 6F), suggesting that FXR1 was at least partially involved in HNRnpa2b1 stabilization.

**Fig 7:**
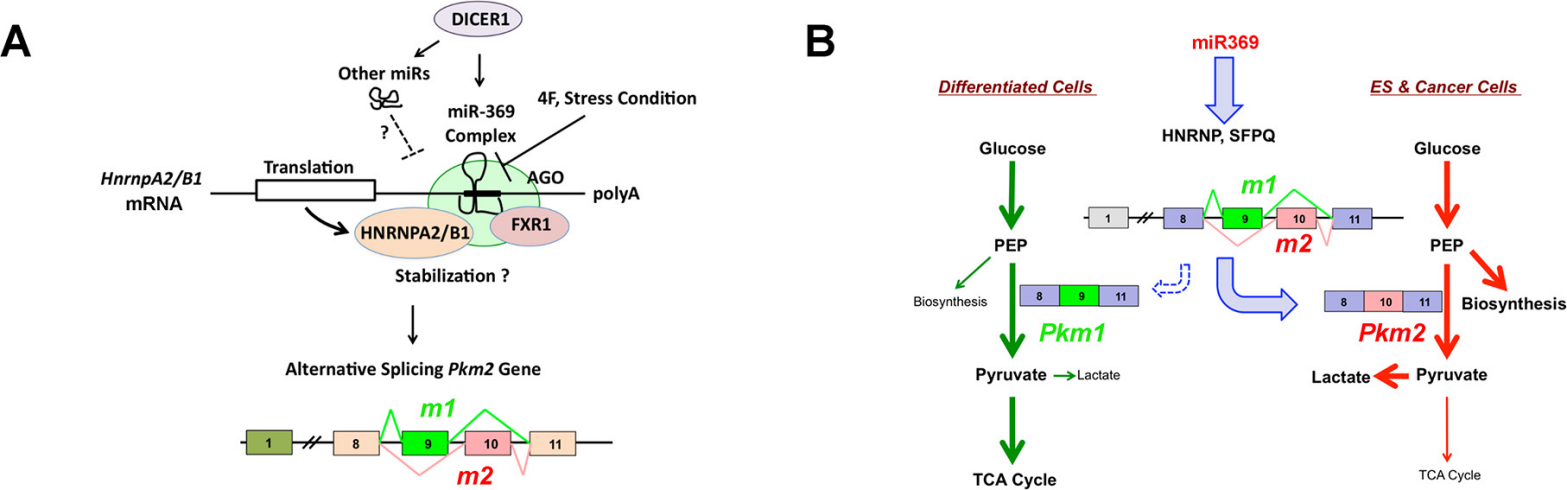
Schema of this study. A) Proposed schema: miR-369 recruits the AGO complex and exerts a unique function on the 3′-UTR of HNRNPA2/B1 mRNA. B)The stabilized HNRNPA2B1 contributes to inducing alternative splicing of PKM1 (exon 9) and PKM2 (exon 10). The latter would be involved in anaerobic glycolysis as in ESCs and cancer cells. PKM1 plays a role in the induction of oxidative phosphorylation in differentiated somatic cells [29].

Given that HNRnpa2/b1 interacts with the double-stranded small cRNA at promoter regions of p21WAF1/CIP1/CDKN1A [35], we assessed how HNRnpa2b1 controls post-transcriptional regulation in a sequence-specific manner in the RISC 3’-UTR. HNRnpa2/b1 was co-immunoprecipitated with AGO in the presence of miR369 in *Dicer1*-deficient conditions. Based on this finding, we were interested in determining whether miR-369 could be involved in the translational stability of the 3’-UTR of hnRnpa2/b1 mRNA. Since this could lead to stabilization of post-transcriptional regulation and translation enhancement, we studied hnRnpa2/b1-transfected ADSCs with CHX or ACD under *Dicer*-deficient and miR-369-sufficient conditions. Immunoblot analysis demonstrated that the replenishment of mature miR-369 in *Dicer* knockout cells increased hnRnpa2/b1 in a time-dependent manner after CHX treatment; but the results were unclear after ACD treatment, suggesting that hnRnpa2/b1 was stabilized by miR-369 sufficiency (Fig A, B in S2 File.). The schema is summarized in Fig 7A and 7B.

## Discussion

The present study reveals a novel mechanism of miR-369 s in the imprinted genomic region at chromosome 12F of mice, of which chromatin modification plays a critical role in the qualification of PSCs, such as in germ line transmission [26]. Although miRs are thought to largely mediate translational repression and mRNA degradation in many somatic cells [15], recent studies have revealed another unique function of miR in the early stages of development since in early embryos and oocytes, miR function is globally suppressed [36, 37]. In contrast to somatic cells, endogenous miRs in oocytes and early embryos were poor at repressing the translation of mRNAs [37], and were unable to localize to P body-like structures that contain RNA-binding proteins [36]. The present study supports the notion that miRs exert distinct biological functions in embryonic cells compared to their function in somatic cells, and maternally expressed miR-369 at chromosome 12qF plays a role in the stabilization of target products under certain circumstances in early embryos. The stabilizing effect of miR-369 is observed not only for HNRNPA2B1 as shown here, but also for the TNF receptor [16]; given that both targets of miR-369 involve the Fxr1 protein, suggesting a shared feature in meiotic cells. Sequencing of small RNA fractions indicates the existence of miR-369 in oocytes, suggesting that miR-369 may play a role in the establishment of glucose flux in early embryonic cells, before transcription from the fertilized genome has reached a significant level and miR functions are globally suppressed.

In summary, the present data suggest that the physiological response in the genomic conserved imprinting region determines cellular metabolism via miR-369 mediated stabilization of translation [16], which switches *Pkm* splicing in the early stages of differentiation. The current findings also link epigenetic networks with metabolism via stress-inducible miRs. The sequence analysis indicates that the miR-369 region at chromosome 12qF of mouse is highly conserved in humans at chromosome 14q32.2-q32.31. Further studies with synthesized nucleotides mimicking miR-369 may open possibilities for tissue engineering and provide novel therapeutic targets, drug screening, and further therapeutic approaches for degenerative disorders by modulating terminal differentiation.

## Supporting Information

S1 File Compared the genome sequence between human and mouse. Mouse chromosome 12qF encompassing the imprinted *Dlk1-Dio3* gene cluster. Data made available on December 2011 (NCBIm38/mm10) were used for mapping. (Fig A) Human chromosome 14q32.2–q32.31 within the analogous *DLK1-DIO3* gene cluster. Human *RIAN* and *MIRG* genes do not locate, while human miR-369 locate within a corresponding region. Available data on February 2009 (NCBIGRch37/hg19) were used for mapping. (Fig B) *In silico* cloning for human genes analogous to mouse *Rian3* and *Mirg*. To search for a homologous human gene to mouse *Rian*, a 57-kb genomic region (−14 kb to +43 kb) around miR-370 of mouse (110,856,468–110,856, and 546 on NCBI M37/mm9) and corresponding human regions (101,377,476–101,377, and 554 on NCBI GRch37/hg19) were aligned using eBioX (http://www.ebioinformatics.org/). Red dots indicate that a homologous region is discontinued, suggesting mouse *Rian* is not conserved in this region (Fig C). To search for a homologous human gene in mouse *Rian* in the extended region, above 57-kb mouse genomic region (−14 kb to +43 kb) around miR-370 (110,856,468–110,856, and 546 on NCBI M37/mm9) were aligned approximately with human 168.5-kbp region encompassing a position −14 kb from miR-370 and miR-369 s (101,531,935–101,532, and 004 on NCBIGRch37/hg19) by using eBioX. Mosaic dots suggest a repeated sequence in humans. No apparent human homologue to mouse *Rian* was found. (Fig D) To search for a homologous human gene to mouse *Mirg*, a 20-kb genomic region (−10 kb to +10 kb) around miR-410 of mouse (109,743,715–109,743, and 795 on NCBI M38/mm10) and corresponding human regions (101,532,294–101,532, and 328 on NCBI GRch37/hg19) were aligned by using eBioX. A discontinuous homology was shown between mouse and human samples, suggesting rearrangement during evolution. The BLAST database (http://blast.ncbi.nlm.nih.gov/Blast.cgi) indicated an EST located in humans (BF376962) in antisense strand, although no coding genes were identified, suggesting a transcription activity of the region. (Fig E).
(TIF)

S2 File Stabilization of post-transcriptional regulation by miR-369. CHX-chased time course experiment of hnRnpa2/b1. Dicer1-KO ADSCs were transfected with miR-369. NCT = scramble RNA control. (Fig A) ACD-chased time course experiment of hnRnpa2/b1; performed similarly to (A). (Fig B).
(TIF)

S3 File PKM1 and PKM2 effects on cell metabolomes. Whole metabolome analysis of Wild type and PKM1-KI ES cells using Mass spectrography. Principal component analysis results indicated a good separation between Wild type and *Pkm1*KI ESCs. Comparison of all metabolites of Wild type and *Pkm1*KI ESCs shown as loading factors (labeled colors). Loading (−1<FL<1) factors indicated the importance of each variable to account for the variability in PC1. When the FL number was small, this was regarded as an important metabolic product of *Pkm1*KI cells. (Fig A, B) Quantum of lactate level for ADSCs and miR-369 over expressed ADSCs conditioned medium. (Fig C).
(TIF)
