# Supplementary figures and images for: Embryonic MicroRNA-369 Controls Metabolic Splicing Factors and Urges Cellular Reprograming
Source: PLoS One. 2015 Jul 15;10(7):e0132789. doi: 10.1371/journal.pone.0132789 (PMC4503752; doi:10.1371/journal.pone.0132789)

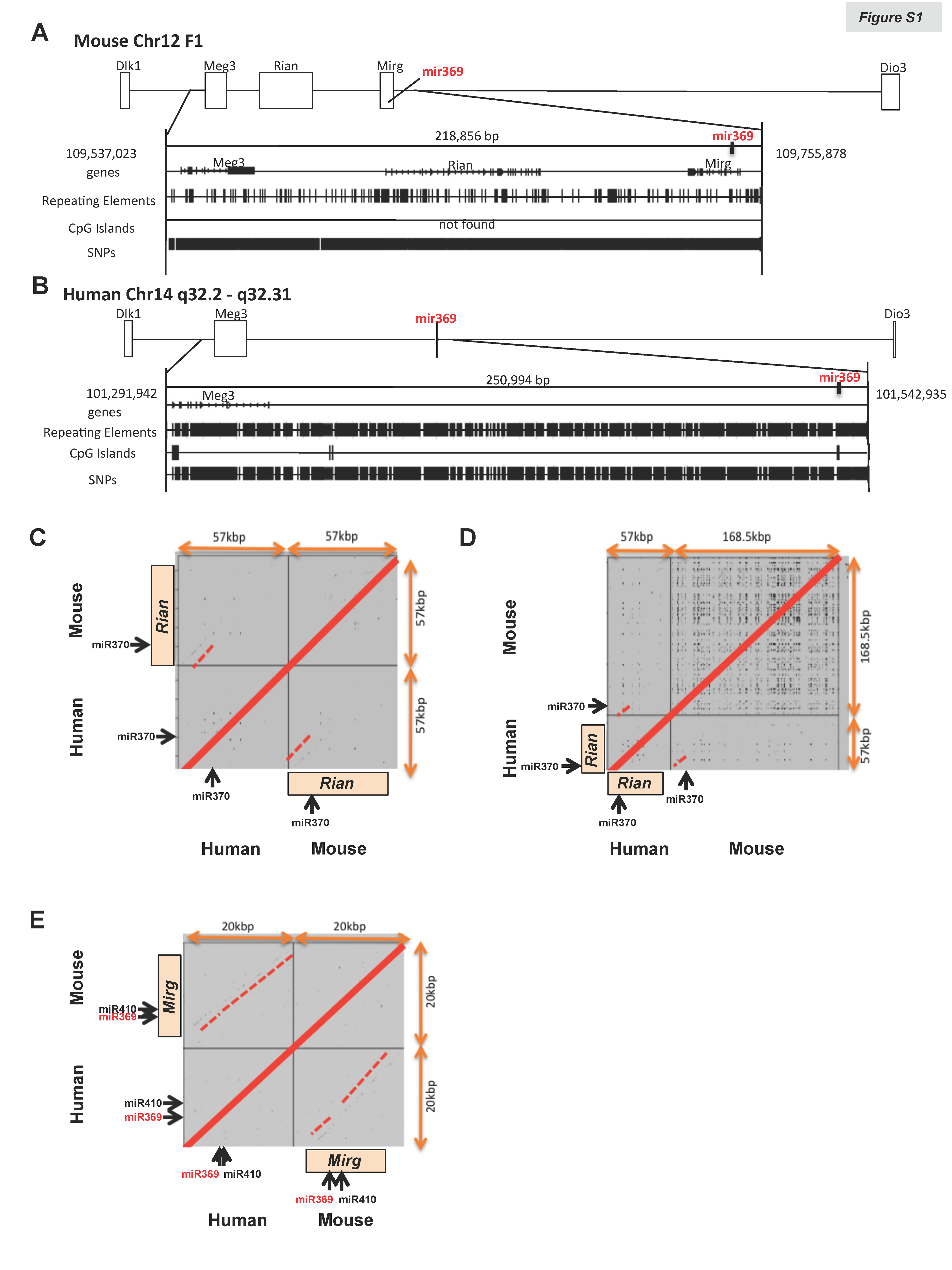

Supplement: S1 File — Mouse chromosome 12qF encompassing the imprinted Dlk1-Dio3 gene cluster. Data made available on December 2011 (NCBIm38/mm10) were used for mapping. (Fig A) Human chromosome 14q32.2–q32.31 within the analogous DLK1-DIO3 gene cluster. Human RIAN and MIRG genes do not locate, while human miR-369 locate within a corresponding region. Available data on February 2009 (NCBIGRch37/hg19) were used for mapping. (Fig B) In silico cloning for human genes analogous to mouse Rian3 and Mirg. To search for a homologous human gene to mouse Rian, a 57-kb genomic region (−14 kb to +43 kb) around miR-370 of mouse (110,856,468–110,856, and 546 on NCBI M37/mm9) and corresponding human regions (101,377,476–101,377, and 554 on NCBI GRch37/hg19) were aligned using eBioX (http://www.ebioinformatics.org/). Red dots indicate that a homologous region is discontinued, suggesting mouse Rian is not conserved in this region (Fig C). To search for a homologous human gene in mouse Rian in the extended region, above 57-kb mouse genomic region (−14 kb to +43 kb) around miR-370 (110,856,468–110,856, and 546 on NCBI M37/mm9) were aligned approximately with human 168.5-kbp region encompassing a position −14 kb from miR-370 and miR-369 s (101,531,935–101,532, and 004 on NCBIGRch37/hg19) by using eBioX. Mosaic dots suggest a repeated sequence in humans. No apparent human homologue to mouse Rian was found. (Fig D) To search for a homologous human gene to mouse Mirg, a 20-kb genomic region (−10 kb to +10 kb) around miR-410 of mouse (109,743,715–109,743, and 795 on NCBI M38/mm10) and corresponding human regions (101,532,294–101,532, and 328 on NCBI GRch37/hg19) were aligned by using eBioX. A discontinuous homology was shown between mouse and human samples, suggesting rearrangement during evolution. The BLAST database (http://blast.ncbi.nlm.nih.gov/Blast.cgi) indicated an EST located in humans (BF376962) in antisense strand, although no coding genes were identified, suggesting a transcription activity of the re [file pone.0132789.s001.tif]

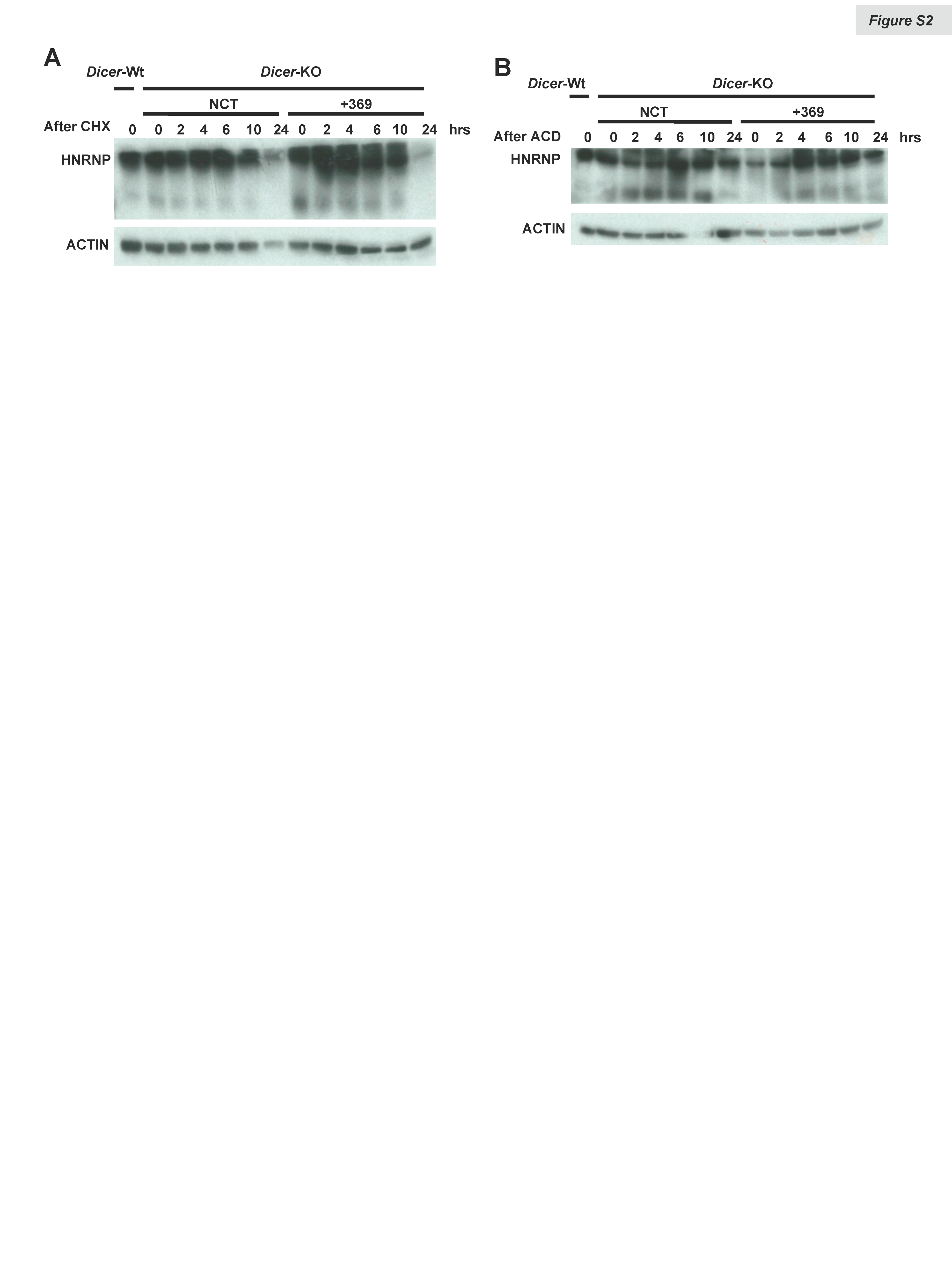

Supplement: S2 File — CHX-chased time course experiment of hnRnpa2/b1. Dicer1-KO ADSCs were transfected with miR-369. NCT = scramble RNA control. (Fig A) ACD-chased time course experiment of hnRnpa2/b1; performed similarly to (A). (Fig B). (TIF) [file pone.0132789.s002.tif]

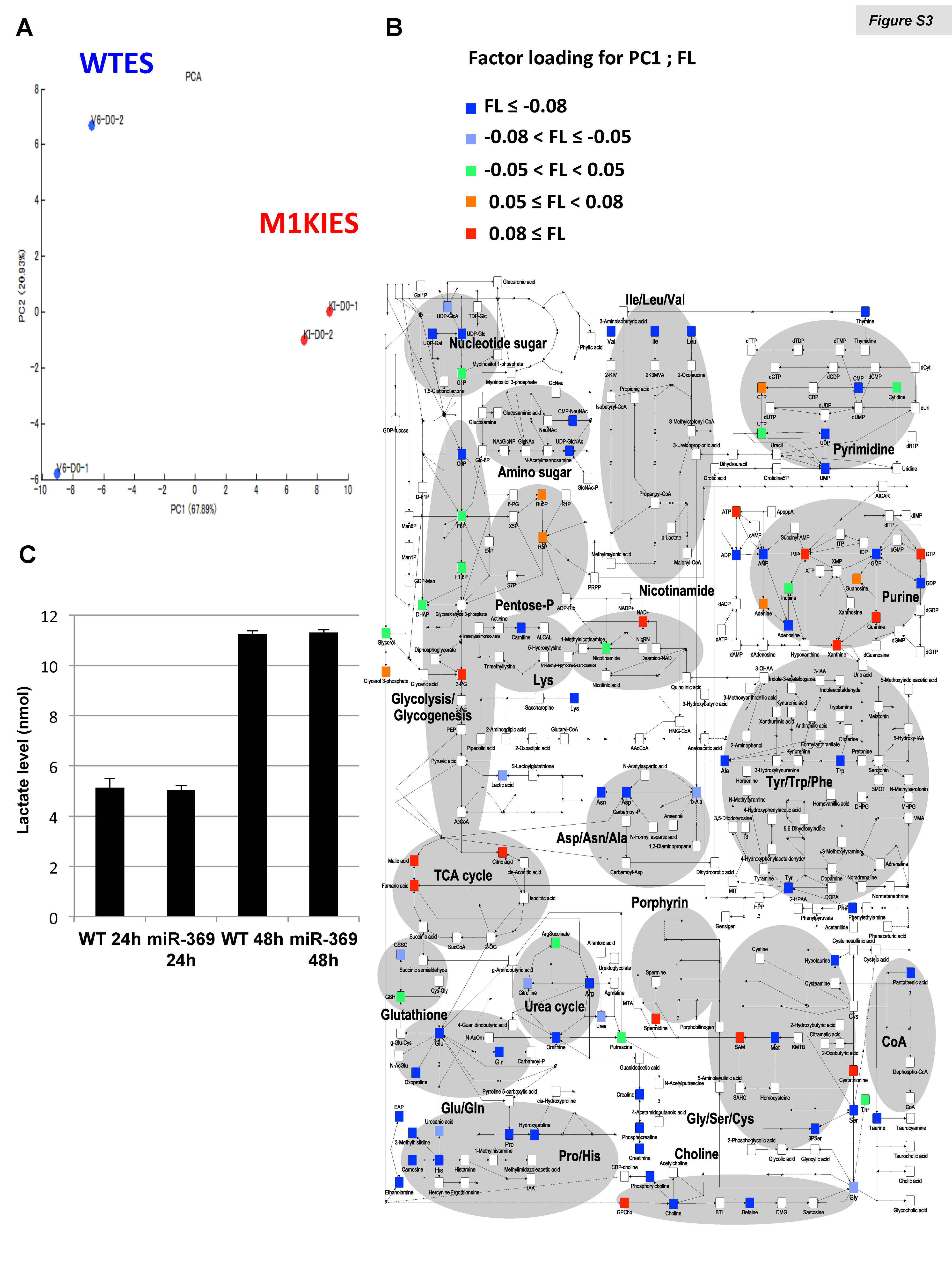

Supplement: S3 File — Whole metabolome analysis of Wild type and PKM1-KI ES cells using Mass spectrography. Principal component analysis results indicated a good separation between Wild type and Pkm1KI ESCs. Comparison of all metabolites of Wild type and Pkm1KI ESCs shown as loading factors (labeled colors). Loading (−1<FL<1) factors indicated the importance of each variable to account for the variability in PC1. When the FL number was small, this was regarded as an important metabolic product of Pkm1KI cells. (Fig A, B) Quantum of lactate level for ADSCs and miR-369 over expressed ADSCs conditioned medium. (Fig C). (TIF) [file pone.0132789.s003.tif]
